# Electron Beam Susceptibility of Enteric Viruses and Surrogate Organisms on Fruit, Seed and Spice Matrices

**DOI:** 10.1007/s12560-021-09463-3

**Published:** 2021-02-10

**Authors:** Sophie Butot, Luca Galbusera, Thierry Putallaz, Sophie Zuber

**Affiliations:** Nestlé Research, Institute of Food Safety and Analytical Science, 1000, 26 Lausanne, Switzerland

**Keywords:** Electron beam, HEEB, LEEB, Enteric viruses, Surrogates, Food

## Abstract

The objective of this study was to use high-energy electron beam (HEEB) treatments to find surrogate microorganisms for enteric viruses and to use the selected surrogates as proof of concept to investigate low-energy electron beam (LEEB) treatments for enteric virus inactivation at industrial scale on frozen blueberries. Six food matrices inoculated with HAV (hepatitis A virus), MNV S99 (murine norovirus), bacteriophages MS2 and Qβ, and *Geobacillus stearothermophilus* spores were treated with HEEB at 10 MeV using 4, 8 and 16 kGy doses. *G. stearothermophilus* spores showed the highest inactivation on all matrices except on raisins, with a dose-dependent effect. HAV reached the maximum measurable log_10_ reduction (> 3.2 log_10_) when treated at 16 kGy on raisins. MNV showed the highest resistance of all tested microorganisms, independent of the dose, except on frozen blueberries. On frozen blueberries, freeze-dried raspberries, sesame seeds and black peppercorns, HAV showed a mean inactivation level in between those of MS2 and *G. stearothermophilus*. Based on this, we selected both surrogate organisms as first approximation to estimate HAV inactivation on frozen blueberries during LEEB treatment at 250 keV using 16 kGy. Reductions of 3.1 and 1.3 log_10_ were measured for *G. stearothermophilus* spores and MS2, respectively, suggesting that a minimum reduction of 1.4 log_10_ can be expected for HAV under the same conditions.

**Supplementary Information:**

The online version contains supplementary material available at 10.1007/s12560-021-09463-3.

## Introduction

Hepatitis A virus (HAV) and human norovirus (hNoV) are responsible for food-borne outbreaks linked to fresh produce, ready-to-eat foods and shellfish worldwide (Alegbeleye et al. 2018; Bosch et al. 2018; Miranda and Schaffner 2019). Outbreaks are generally associated with food that undergoes minimal processing, such as oysters, fresh-cut lettuce, frozen berries and very recently dried dates (Ethelberg et al. 2010; Tavoschi et al. 2015; Thebault et al. 2013; Anonymous 2018). Consumer demands for minimally processed foods with fresh-like quality are on the rise and food processes must adapt to the preferences, acceptance and needs of the consumer (Knorr and Watzke 2019). In this context, innovative approaches are needed to minimize the risk of viral food-borne outbreaks while retaining good food quality. Non-thermal processes, e.g. high hydrostatic pressure (HHP), ultra violet light (UV-C), cold plasma and irradiation, have the potential to achieve this goal (Knorr and Watzke 2019). However, virus inactivation by UV-C light is very efficient in clear liquids such as water (Tree et al. 2005; Wiedenmann et al. 1993), but of limited use on complex food surfaces such as berries (Butot et al. 2018). Virus inactivation by HHP is only applicable to high moisture foods, such as oysters and fruit purees (DiCaprio et al. 2019; Kingsley and Chen 2009). For fragile or dry foods, such as berries, herbs and spices, other technologies need to be explored.

Irradiation appears to be a good candidate technology, but data describing the effectiveness of irradiation on food against viruses, especially HAV, are scarce. Besides X-ray, two major irradiation technologies have been explored, gamma irradiation and high-energy electron beam (HEEB) (Farkas 1998; Moosekian et al. 2012; Pillai and Shayanfar 2017; Ravindran and Jaiswal 2019). Food matrix is likely to provide increased survival for viruses during irradiation, but a major hurdle when performing inactivation studies with viruses on food is the low recovery efficiency which is often encountered, leading to a low maximum measurable log_10_ reduction (Bosch et al. 2018; Butot et al. 2014). For example, at a HEEB dose of 12 kilo Gray (kGy), inactivation of MNV (murine norovirus used as hNoV surrogate) in phosphate-buffered saline (PBS) reached 6.4 log_10_, while on strawberries a maximum reduction of 2.2 log_10_ was observed (Sanglay et al. 2011). Similarly, after a 11.9 kGy treatment in simple medium (DMEM) a 4.25 log_10_ reduction of Tulane virus (TV) was measured, compared to a 2.1 log_10_ reduction after a treatment of 12.2 kGy on whole strawberries (DiCaprio et al. 2016). The *D*_10_ values (dose required to reduce virus titer by 1 log_10_) of rotavirus and poliovirus on lettuce were 1.0 and 2.3 kGy, respectively, suggesting virus-dependent susceptibilities to HEEB (Espinosa et al. 2012). Using gamma irradiation, *D*_10_ values determined for HAV in lettuce and strawberries were 2.7 and 3.0 kGy, respectively (Bidawid et al. 2000), while the HEEB dose required to reduce HAV by 1 log_10_ in whole oysters was 4.8 kGy (Praveen et al. 2013).

A new approach to irradiation processing of food, called low-energy electron beam (LEEB), uses electrons with energies below 300 kilo electron Volt (keV), associated with a very limited penetration ability of around 500 μm (Gryczka et al. 2018; Hayashi et al. 1998; Pillai and Shayanfar 2017). In contrast, HEEB uses electrons with energies between 5 and 10 Mega electron Volt (MeV) which penetrate foods with high water content up to 3.9 cm (Farkas 2006) and has to be carried out at a dedicated irradiation facility with appropriate safety measures. In contrast, the LEEB technology is scalable to continuous processes and can be easily implemented in existing processing lines and offers benefits in cost reduction, environmental performance, and production flexibility (Zhang et al. 2018; Tetra Pak 2019). As such, LEEB is an emerging irradiation technology that performs surface and subsurface decontamination with a minimal influence on food quality (Hertwig et al. 2018). Since microorganisms reside mostly on the surface and subsurface of food if internalized through pores or damaged tissue, the irradiation of the external layer should be sufficient to eliminate food-borne microorganisms (De Lara et al. 2002; Hayashi et al. 1998). For example, Kikuchi et al. recommended LEEB treatment over gamma irradiation for soybean decontamination, because it induces minimum or no quality deterioration, since the electrons do not reach the internal matrix (Kikuchi et al. 2003). Similarly, LEEB was proposed as a method for microbiological decontamination of seeds, as it was shown not to negatively influence germination (Fan et al. 2017). The response of *Bacillus pumilus* spores was found to be very similar when treated with HEEB and LEEB (Tallentire et al. 2010). Another study investigating *Geobacillus* and *Bacillus* spores also showed that the spore inactivation efficiency by LEEB was comparable to that of other ionizing radiations (Zhang et al. 2018). LEEB was also shown to reduce mould spores even on challenging food surfaces such as raisins (Etter et al. 2018). Inactivation of enveloped viruses such as influenza A in liquids has been demonstrated (Etter et al. 2018), but no data are available on the effect LEEB has on non-enveloped enteric viruses such as HAV and hNoV. The only industrial scale system currently available to the food industry is the Bühler AG Laatu system. As this system is an open system, it does not allow safe handling of pathogens and non-pathogenic surrogates are needed to estimate virus inactivation by LEEB.

The objective of this study was first to use HEEB treatments to find surrogate microorganisms for enteric viruses (HAV and hNoV) which are easy to grow to high titers and use at industrial scale such as bacterial spores or bacteriophages and second to use the surrogate organisms as proof of concept to investigate LEEB treatments for enteric virus inactivation at industrial scale using frozen blueberries as a model matrix.

## Materials and methods

### HEEB Irradiation Treatment and Dose Evaluation

HEEB treatments (10 MeV) were performed at the LEONI Studer AG irradiation centre (Daeniken, Switzerland). Inoculated food samples were treated at three radiation doses, 4, 8 and 16 kGy, to define critical dosages and screen for adequate surrogates. Pretests were performed for the different food matrices used in this study to verify dose distribution within the load. Based on the results, samples were packaged in 50 mL Falcon tubes with a maximum diameter of 3 cm which ensured equal irradiation doses throughout the samples. The absorbed doses were measured using alanine pellets (Aerial CRT, France) which were treated together with the samples (nine independent treatments performed on three separate days) and the doses were calculated by comparison with a standard calibration curve established by Aerial CRT onsite at LEONI Studer AG irradiation centre and traceable to NPL standard. The following mean values and standard deviations were obtained: 3.91 ± 0.03, 7.89 ± 0.14 and 15.85 ± 0.33 kGy. The results showed that the values for HEEB are close to the nominal doses, and thus, the nominal values are used in the graphs.

### LEEB Irradiation Treatment and Dose Evaluation

LEEB treatments were performed with Laatu designed by Bühler AG (Uzwil, Switzerland). Inoculated frozen blueberries were treated twice with 250 keV to reach a nominal dose of 16 kGy. Samples were conveyed via an inlet channel and feeder table which control the throughput and distribution of the product. The berries were treated during free-fall through the treatment zone which consists of two LEEB lamps facing each other (https://digital.buhlergroup.com/laatu/). The dose was measured with Riso B3 radiochromic film sensor provided by Riso High Dose Reference Laboratory (HDLR) (Roskilde, Denmark). Due to the practical limitations of placing a dosimeter on the frozen blueberries, a B3 film strip of 230 mm × 20 mm was attached along a polymer plate covering the entire width of the treatment zone. The dosimeters were exposed to the same beam conditions (voltage, current) as the inoculated frozen blueberries. The dosimetry system (including radiochromic film, sensor measurement software and calibration) is traceable to international standards in line with ISO/ASTM 51261 (ISO 2013). Dose measurements using Riso B3 films determined a mean surface dose value of 15.51 ± 1.31 kGy which is close to the nominal dose, and thus, the nominal value is used in the graph.

### Food Matrices and Pretreatments

Six different food matrices were used in this study. Pumpkin, and sesame seeds were kindly provided by the German Institute of Food Technologies (DIL) (Quakenbrück, Germany), black peppercorns, fresh blueberries, freeze-dried raspberry flakes and raisins were purchased at Sabater (Spain), a local distributor (Lausanne, Switzerland), Chaucer (UK) and Mariani Packing (USA), respectively. X-ray pretreatments (25–50 kGy) of the matrices to kill background microflora before inoculation with target organisms were performed at Synergy Health Daeniken AG (Daeniken, Switzerland).

### *Geobacillus* Spore Suspension

Ready to use suspension of *Geobacillus stearothermophilus* (ATCC 7953) spores, containing 7.7–8.7 log_10_ colony forming units (CFU)/mL of viable spores, was purchased from Merck Millipore (Burlington, USA) and was used to inoculate the different food matrices.

### Viruses and Preparation of Suspensions

The cytopathogenic HAV strain HM-175 (ATCC VR-1402), the murine norovirus strain MNV S99 used as proxy for hNoV and provided courtesy of the Friedrich-Loeffler-Institut in Germany (Mueller 2007) were propagated, assayed and titrated on FRhK-4 cells (ATTC CRL1688) and RAW 264.7 cells (ATCC TIB-71), respectively, as described previously (Butot et al. 2018). Viral stock titers were 6.6 and 7.6 log_10_ 50% tissue culture infective dose (TCID_50_)/mL for HAV and MNV, respectively. MS2 bacteriophage (ATCC 15597-B1) was propagated, assayed and enumerated in *Escherichia coli* K12 (ATCC 23631), as described previously (Butot et al. 2018). The Qβ bacteriophage (ATTC 23631-B1) was propagated, assayed and enumerated using the same protocols as for MS2. The MS2 and Qβ stock concentrations were 11.3 and 10.1 log_10_ plaque forming units (PFU)/mL, respectively.

### Inoculation of Food Matrices with Bacteria and Viruses

Suspension (100 μL) of bacterial spores, viruses or bacteriophages were spotted (droplets of 5.2 ± 0.4 μL) directly on the food surface of the seven selected food matrices (pumpkin, and sesame seeds, peppercorns, fresh blueberries, freeze-dried raspberries and raisins) using a technique described previously (Delbeke et al. 2015). For the HEEB treatment, 10 g of each food matrix were inoculated with bacterial spores, viruses or bacteriophages, respectively, except for the freeze-dried raspberries, for which 5 g was used. The contaminated samples were allowed to dry in a biosafety cabinet at room temperature for 1 h, before being packaged in a 50 mL tube and subjected to the HEEB treatment. The inoculated fresh blueberry samples were frozen at − 20 °C after the packaging. Following the same approach, blueberry samples were inoculated with *G. stearothermophilus* and MS2 bacteriophages and frozen prior to the LEEB treatment.

### Recovery of Contaminants from Food Matrices

To recover inoculated spores of *G. stearothermophilus* from matrices, 5 or 10 g sample was aseptically transferred to a stomacher filter bag, diluted 1:10 in buffered peptone water and stomached for 1 min. From this initial suspension, 10 mL was submitted to a heat treatment at 80 °C for 10 min to allow the spores to germinate. After the heat treatment, tubes were cooled down in cold water. Serial tenfold dilutions in tryptone salt solution were performed and plated on Plate Count Agar supplemented with 1 g/L of soluble starch and incubated at 55 °C ± 1 °C for 48 h ± 4 h. The limit of quantification (LOQ) was 2.00 log_10_ CFU/10 g for all food matrices except for the freeze-dried raspberries, for which the LOQ was 1.98 log_10_ CFU/5 g.

HAV and MNV were recovered from 5 or 10 g samples using the ISO 15,216 virus extraction method (soft fruits protocol) with slight modifications: no added process control, as untreated samples were analyzed to determine recovery rate and the chloroform/butanol clarification step was not performed (ISO 2017). Before enumeration, concentrated samples were decontaminated by sequential filtering through 0.45 μm and then 0.22 μm spin centrifuge tube filters (Corning, New York) pretreated with 300 μL of phosphate-buffered saline (pH 7.2 ± 0.2) containing 10% fetal calf serum followed by TCID_50_ titration as previously described (Butot et al. 2018). The LOQ was 1.05 log_10_ TCID_50_/10 g for all food matrices except for the freeze-dried raspberries, for which the LOQ was 1.35 log_10_ TCID_50_/5 g as the pellet obtained after the polyethylene glycol precipitation was too viscous and therefore resuspended in 1 mL instead of 0.5 mL.

MS2 and Qβ were recovered from 5 or 10 g samples using the protocol described previously (Butot et al. 2018). Briefly, the sample was transferred in a filter bag containing 50 mL of buffer (100 mM Tris, 50 mM glycine, 3% (m/v) beef extract and 50 mM MgCl_2_ and adjusted to pH 9.5 with NaOH solution) and stomached for 1 min. Serial dilutions (tenfold) were prepared and virus titer was quantified using ISO method 10705–1:1995(E), Annex C (ISO 1995). The LOQ was 2.78 log_10_ PFU/10 g for all food matrices except for the freeze-dried raspberries, for which the LOQ was 2.74 log_10_ PFU/5 g.

For all matrix/strain combinations, untreated inoculated triplicate samples were analyzed to determine the log_10_ reductions, the maximum measurable log_10_ reduction calculated as log_10_ (LOQ/*N*_*0*_) and the recovery rates and un-contaminated triplicate samples were analyzed as negative controls following the procedures described above.

### Statistical Analysis

Reductions in spore and infectious viral particle counts (inactivation) were calculated as log_10_ (*N*_*x*_/*N*_0_), where *N*_*x*_ is the spore count or the viral titer recovered from treated food matrices and *N*_0_ is the initial count or titer recovered from untreated food matrices (mean of three replicates). Values of *N*_*x*_ below the LOQ were entered as being at that limit and this leads the log_10_ reduction to reach the maximum measurable log_10_ reduction.

A *t* test with a significance level at 0.05 and without correction for multiplicity of test was used to test whether the mean log_10_ reduction of the replicates in one condition was statistically different from the mean in another condition. Furthermore, it is also important to understand if the single measurements are consistently higher or lower. Thus, for each condition we also computed a prediction interval, corresponding to the interval where 95% of log_10_ reductions measured in future experiments are expected to fall. If the prediction intervals of two conditions are non-overlapping, we can be confident that not only the mean log_10_ reductions are different, but also that the single observations in future experiments are going to be well separated.

In order to compute the *t* tests and the prediction intervals, the variance inside each condition is needed. However, as the maximum measurable log_10_ reduction was reached in some conditions, the measured values are not representative of the real ones and must be discarded in the computation of the variance. This leads to having conditions with very few points (possibly zero) from which to estimate the variance. To solve this issue, we assumed that the variance in the log_10_ reductions of a microorganism in a given matrix is independent from the dose. This allows us to use ANOVA on the values above maximum measurable log_10_ reduction to compute a pooled variance, common to all the measurements of a given microorganism in a given matrix. Notice that since the width of the prediction interval depends only on the variance of the observations, the prediction intervals have the same width for all the doses of a given microorganism and matrix. Finally, the mean log reductions computed in the presence of values at the maximum measurable log_10_ reduction are just a lower bound, since the real log reductions might be higher. This means that the presence of values at the maximum measurable log_10_ reduction can hide the effectiveness of the treatment, since the real mean log reduction might actually be bigger than the estimated one. However, this does not affect the size of the prediction intervals, which are computed using only values above the maximum measurable log_10_ reduction.

We notice that the overall variability of the data is determined by different sources, for example, a variability in the recovery rates and in the irradiation values, and a biological variability in the response of the microorganisms. Therefore, the variance computed as described before takes into account all these sources of variability, that are also likely to be found when applying the irradiation technology to real production samples.

All statistical analyses were performed with R v3.6.1 (R-Core Team 2019).

## Results

### Recovery Efficiencies

To evaluate the efficiency of the methodologies employed, the recovery of each microorganism was determined for all matrices tested as the ratio, in percentage, of *N*_0_ which corresponds to the initial count recovered from untreated matrices and the calculated theoretical count using the stock concentration and the volume inoculated (Table 1). *G. stearothermophilus* was recovered efficiently with mean values ranging between 46.61 and 100.79% for all matrices, except for the freeze-dried raspberries where a rate of 25.72% was obtained. On the opposite, the bacteriophages and the viruses showed lower recovery rates. The bacteriophages were recovered with efficiencies ranging from 0.03 to 9.92% for MS2 and 0.03–17.76% for Qβ. Matrix effects were observed but no correlation was found between the two bacteriophages. Recovery rates above 1% were obtained with sesame seed and frozen blueberry matrices for MS2 and with sesame seed, pumpkin seed and raisin samples for Qβ. For viruses, the recovery rates oscillated from 0.12 and 4.22% for HAV and from 0.01 to 16.24% for the hNoV proxy MNV. Recoveries above 1% for HAV were reached only on raisins and for MNV only on sesame seeds and peppercorns. The freeze-dried raspberries showed low recoveries for all the microorganisms (Table 1). In addition, this food product induced a cytotoxic effect on the RAW 264.7 cells, making it impossible to report inactivation results for MNV. However, this effect was not observed on the FrhK4 cells used to measure the infectivity of HAV.

**Table 1:** Recovery efficiencies of each microorganism–matrix combination expressed in percentage (%)

	*Geobacillus*	HAV	MNV	MS2	Qβ
Frozen blueberries	100.79	0.65	0.01	1.59	0.04
Freeze-dried raspberries	25.72	0.40	–	0.03	0.05
Raisins	92.86	4.22	0.06	0.09	2.97
Peppercorns	82.54	0.89	16.24	0.80	0.03
Sesame seeds	46.61	0.46	1.35	9.92	17.76
Pumpkin seeds	63.92	0.12	0.60	0.06	7.26

– the missing value could not be determined due to technical issues

The recovery efficiency is an important parameter as it has an impact on the maximum measurable log_10_ reduction. Indeed, the lower the recovery, the lower the *N*_*0*_ and the maximum measurable log_10_ reduction will be. As an example, 5.45 log_10_ TCID_50_ of HAV were inoculated on 5 g freeze-dried raspberries, but with a recovery efficiency of 0.40%, only 3.04 log_10_ TCID_50_/5 g of HAV were enumerated on the samples not treated (*N*_*0*_). Therefore, taking into consideration this *N*_*0*_ value and the LOQ of 1.35 log_10_ TCID_50_/5 g, the maximum measurable log_10_ reduction was 1.69 log_10_.

We notice that a low recovery rate limits the maximum measurable log_10_ reduction, therefore decreasing the power of detecting strong reductions. However, even for the treatments where the low recovery leads to values below the LOQ, meaningful conclusions can still be drawn, as will be discussed in the following sections. Also, the low variability rates can increase the variance of the measured effects. However, as explained in “Materials and Methods” section, the computation of the *t* tests and of the prediction intervals take into account also any variation in the recoveries.

### Reductions of HAV and MNV By HEEB Treatment on Different Food Matrices

**Fig. 1:**
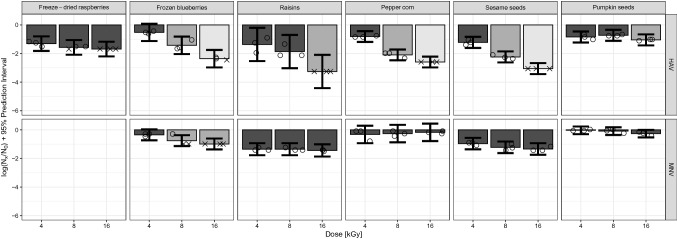
Mean inactivation of HAV and MNV on freeze-dried raspberries, frozen blueberries, raisins, pepper corns, sesame seeds and pumpkin seeds treated with HEEB at 4, 8 and 16 kGy. Different shadings show significant differences in the mean log_10_ reductions between dosages within each microorganism–matrix combination. Bars represent 95% prediction intervals. Open circles represent single values, crosses represent single values below the maximum measurable log_10_ reduction. To compute the mean log reduction, both circles and crosses have been taken into account. For this reason, in the presence of values below the maximum measurable log_10_ reduction, we only have a lower bound on the mean reduction. For the prediction intervals, only the circles have been considered, and a common variance has been computed for a given microorganism-matrix combination. Therefore, all the prediction intervals for a given combination have the same widths, and they are not affected by having values below the maximum measurable log_10_ reduction. Supplementary Table 1 contains the raw data used to generate Fig. 1

Inactivation values of HAV and MNV inoculated onto the different model food matrices (fruit, spice and seed) are shown in Fig. 1. The results are shown in log_10_ reduction with 95% prediction intervals (not confidence intervals) to better represent the “minimal” effect of a certain treatment condition. A measure obtained from a future experiment will fall on this interval with a probability of 95%. The highest reduction of HAV was observed when inoculated on raisins (Fig. 1). On this matrix at 8 kGy, a mean reduction of 1.9 log_10_ was measured, ensuring a minimal reduction of only 0.7 log_10_, according to the 95% prediction interval. At 16 kGy the maximum measurable log_10_ reduction was reached (> 3.2 log_10_), corresponding to a minimal reduction which is at least 2 log_10_ as given by the prediction interval. Notice that we talk about minimal reduction, because the real mean reduction might be bigger than the estimated one, as the maximum measurable log_10_ reduction has been reached for all the three replicates (this does not affect the size of the prediction intervals, which are computed only from values above the maximum measurable log_10_ reduction). A dose-dependent inactivation of HAV was also observed on frozen blueberries with mean reduction values of 0.5, 1.4 and 2.4 log_10_ at 4, 8 and 16 kGy, respectively. For comparison with raisins, the minimal log_10_ reduction at 16 kGy on frozen blueberries was estimated at 1.75 log_10_. The reductions of HAV inoculated on freeze-dried raspberries subjected to the three HEEB dosages of 4, 8 and 16 kGy appeared not significantly different from each other (bars of the same colour for all three dosages), but this may be an artefact which could be overcome if we had a better extraction efficiency allowing to see a greater maximum measurable log_10_ reduction than 1.7 log_10_ at 16 kGy. A significant dose-dependent inactivation of HAV subjected on HEEB treatment of 4, 8 and 16 kGy was seen on peppercorns and sesame seeds, with mean HAV reductions of 2.1 and 2.2 log_10_ when treated at 8 kGy, respectively. At 16 kGy the maximum measurable log_10_ reduction was reached on both matrices, corresponding to 2.6 log_10_ for peppercorns and 3.1 log_10_ for sesame seeds. The reductions of HAV inoculated on pumpkin seeds were lower than the reductions observed on sesame seeds (a mean inactivation of only 1.1 log_10_ at 16 kGy) which may indicate complex matrix effects and show that predictions of inactivation are risky, even for similar food matrices.

Compared to HAV, MNV showed a higher resistance to HEEB on all matrices, except for frozen blueberries where maximum measurable log_10_ reductions were reached in most cases, preventing a meaningful comparison (Fig. 1). This highlights the high resistance of MNV to irradiation by HEEB on food matrices (complementary Table 1). The inactivation was lowest on pumpkin seeds and peppercorns with mean inactivation values showing no significant differences among the three dosages applied and ranging from 0.03 to 0.32 log_10_. MNV inoculated on raisins and sesame seeds did not show significant dose-dependent differences either, with reductions ranging from 1.35 to 1.44 log_10_ on raisins and 0.96 and 1.34 log_10_ on sesame seeds. A significantly higher inactivation at a higher HEEB dose could only be measured for MNV on frozen blueberries with mean log reductions of 0.35, > 0.76 and > 1.18 log_10_ for treatments at 4, 8 and 16 kGy, respectively.

### Reductions of Selected Surrogate Organisms By HEEB Treatment

**Fig. 2:**
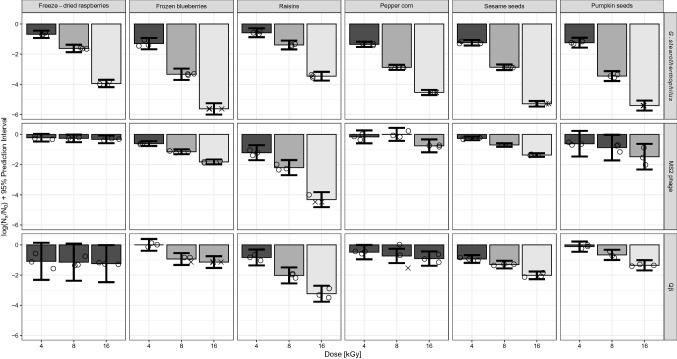
Mean inactivation of *G. stearothermophilus* spores, MS2 and Qβ bacteriophages on freeze-dried raspberries, frozen blueberries, raisins, pepper corns, sesame seeds and pumpkin seeds treated with HEEB at 4, 8 and 16 kGy. Different shadings show significant differences in the mean log_10_ reductions between dosages within each microorganism-matrix combination. Bars represent 95% prediction intervals. Open circles represent single values, crosses represent single values below the maximum measurable log_10_ reduction. Notice that since a common variance has been computed for a given microorganism-matrix combination, all the prediction intervals for that combination have the same width. Supplementary Table 2 contains the raw data used to generate Fig. 2

Inactivation values of the three surrogate organisms (spores *G. stearothermophilus* and bacteriophages MS2 and Qβ) inoculated onto the different model food matrices (fruit, spice and seed) are shown in Fig. 2. MS2 and Qβ both showed a high resistance to HEEB treatments compared to *G. stearothermophilus*, with mean reductions generally lower than 1.5 log_10_. The only exceptions were raisins, where both bacteriophages showed reductions close to those for *G. stearothermophilus*. We noticed that for some matrices, the prediction intervals at two different irradiation doses (error bars) were overlapping, meaning that the doses do not have a statistically different effect. That is, we cannot conclude that future measurements at the higher dose will have a higher log reduction compared to the lower dose. The overlapping of prediction intervals can happen even when the means are statistically different (the bars have a different shade of grey), as for example for Qβ in sesame seeds treated at 4 and 8 kGy. This shows that, although the mean reductions can be different at two different irradiation doses, for single future measurements we cannot conclude that the reduction is lower at 4 kGy compared to 8 kGy.

On sesame seeds, however, MS2 showed a clear lower reduction compared to Qβ, both considering the mean and the prediction interval (Supplementary Table 2). Given the similarities between the two bacteriophages, and the fact that compared to Qβ, MS2 showed a higher recovery and a dose-dependent reduction on frozen blueberries, we chose to focus only on MS2 as candidate surrogate for HAV and hNoV for the LEEB study. This choice is especially relevant for HAV, as on all food matrices and doses, HAV showed a mean reduction which was either higher or non-statistically different than the mean reduction for MS2 (Supplementary Table 3).

*Geobacillus stearothermophilus* showed a dose-dependent inactivation on all tested matrices with a generally higher inactivation than the tested viruses, for both mean reductions and prediction intervals, except on raisins (Fig. 2). On frozen blueberries, for example, a 1.3, 3.3 and > 5.6 log_10_ reduction was measured for HEEB treatments at 4, 8 and 16 kGy, respectively. In comparison, at 16 kGy on the same matrix, 2.4 log_10_ and 1.8 log_10_ reductions were measured for HAV and MS2, respectively (Figs. 1, 2). Similarly, on sesame seeds, pumpkin seeds and peppercorns at 4, 8 and 16 kGy, HAV showed a lower mean inactivation level compared to *G. stearothermophilus* (Figs. 1, 2). This intermediate ranking of HAV between *G. stearothermophilus* spores and MS2 determined by HEEB was ideal to select both surrogate organisms as first approximation to estimate HAV inactivation during LEEB treatments of frozen blueberries.

### Inactivation of *G. stearothermophilus* Spores and MS2 During LEEB on Frozen Blueberries

**Fig. 3:**
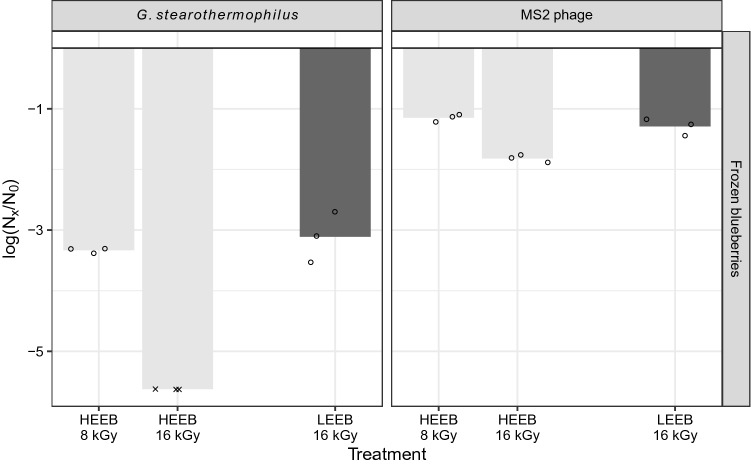
Mean inactivation of *G. stearothermophilus* spores, HAV and MS2 bacteriophage on frozen blueberries treated with LEEB at 16 kGy in comparison with corresponding reduction generated by HEEB at 8 and 16 kGy. Open circles represent single values, crosses represent single values below the maximum measurable log_10_ reduction

*Geobacillus stearothermophilus* spores and MS2 bacteriophage on frozen blueberries were treated in the industrial Bühler AG Laatu system, as a proof of concept for validation of HAV and hNoV inactivation by LEEB. In Fig. 3, the LEEB results obtained from this experiment are compared with the data generated by HEEB. As the LEEB experiment was a proof of concept with a low amount of data, we did not carry out statistical analysis on these data points. Mean reductions of 3.1 log_10_ and 1.3 log_10_ were measured after the LEEB surface treatment for *G. stearothermophilus* spores and MS2, respectively (Fig. 3). These values were similar to the log_10_ reductions determined for both organisms in the HEEB trials at 8 kGy (3.3 log_10_ for *G. stearothermophilus* spores and 1.1 log_10_ for MS2). The similarity of the log_10_ reductions of the surrogates observed on frozen blueberries after a LEEB treatment at 16 kGy and a HEEB treatment at 8 kGy, suggests that if HAV underwent a LEEB treatment at 16 kGy, we might expect a reduction similar to the one determined for the HEEB treatment at 8 kGy (Fig. 1). Thus, we expect to see a log_10_ reduction of 1.4 on frozen blueberries for HAV treated by LEEB at 16 kGy.

## Discussion

The results of the present study represent a proof of concept for validation of HAV and hNoV inactivation by LEEB, using the industrial Bühler AG Laatu system, the only industrial scale system currently available to the food industry. As this system is an open system not allowing safe handling of pathogens, the HEEB technology was used with inoculated and packaged food matrices to compare reduction of HAV and hNoV with potential surrogates. The HEEB trials generated new data on the effect electron beam irradiation has on different viruses-food matrix combinations. Overall, this study demonstrates the high resistance of HAV to electron beam irradiation and an even higher resistance of MNV (Fig. 1). As different amino acids vary in their susceptibility to irradiation, crosslinking of proteins during irradiation may damage portions of the capsid involved in receptor binding to varying degrees and may explain differences in susceptibility observed between different viruses (Hume et al. 2016; Stewart 2001). A comparison with hNoV is needed in the future to determine which hNoV surrogate can reliably be used as surrogate to evaluate the effect of electron beam irradiation. Using the porcine gastric mucin magnetic bead (PGM-MB) binding assay, the level of hNoV and TV RNA decreased by > 1 log_10_ and approximately 2 log_10_, respectively, following a 12.2 kGy HEEB treatment on whole strawberries (DiCaprio et al. 2016). In comparison, at 8 kGy for MNV our results reveal log_10_ reductions of 0.76 and 1.35 log_10_ on frozen blueberries and raisins, respectively. As shown by Ettayebi and coauthors using the stem cell derived human enteroids assay, exposure to gamma irradiation of hNoV GII.3 and GII.4 stool suspensions inactivated the viruses, but at this point in time no data are available with this system on food matrices (Ettayebi et al. 2016).

As reported by others, spores of *G. stearothermophilus* are highly resistant against irradiation and showed a higher resistance during LEEB than the spores of *B. pumilus* which is often suggested to be the biological indicator for irradiation sterilization (Van Gerwen et al. 1999; Zhang et al. 2018). Therefore, *G. stearothermophilus* spores were included in this study to compare their resistance to electron beam with the one of HAV. We observed a higher susceptibility of *G. stearothermophilus* to HEEB compared to HAV (Figs. 1, 2). The data generated in this study for *G. stearothermophilus* is new and valuable for future industrial validation studies. It cannot be directly compared with the data present in the literature, as most studies have determined *D*_10_ values for electron beam treatments of *B. pumilus* and *B. subtilis* and to a lesser extent *G. stearothermophilus* spores not inoculated onto any food matrix (Tallentire et al. 2010; Zhang et al. 2020, 2018). Nevertheless, the *D*-value of 3.1 kGy at 200 keV measured by Zhang and coauthors corresponds to a 2.6 log_10_ reduction at 8 kGy which is similar to the reduction of 2.87 log_10_ we measured on peppercorn and sesame seeds. Additionally, the food matrix can have opposite effects on different microorganisms. For example, on raisins *G. stearothermophilus* showed a lower inactivation than on the other matrices. Viruses, on the other hand, generally showed higher inactivation on raisins compared to the other matrices, showing that extrapolation of effects from one matrix to another should not be attempted.

Low performance of the methods used to recover the viruses and the bacteriophages impacted only a small part of the results, as the maximum measurable log_10_ reductions were in most cases sufficient to draw meaningful conclusions. *G. stearothermophilus* spores showed good recoveries on every matrix, whereas enteric viruses and bacteriophages displayed poor recovery rates. For enteric viruses, such as HAV and MNV, this was expected as the methods available for the detection of viruses are well known for their complexities and low recovery efficiencies (Li et al. 2018; Mäde et al. 2013; Perrin et al. 2015). In ISO 15,216, the minimal recovery efficiency required is 1% but this requirement is often not reached (Li et al. 2018). For example, Perrin and coauthors reported a recovery of 0.5% of MNV on raspberries and Hida and coauthors reported MNV recovery efficiencies of 0.06% and 0.41% on lettuce and grapes, respectively (Hida et al. 2018; Perrin et al. 2015). The low recovery efficiencies obtained with the bacteriophages were more surprising as the method used is in principle simple and easy to apply. Shim and coauthors showed a correlation between the stronger adhesion and the lower recoveries of MS2 on PVC, therefore one hypothesis of this low performance could be a strong adhesion of the bacteriophages on the matrices, impacting the recovery rates (Shim et al. 2017). One explanation of this stronger adhesion could be the time between the inoculation and the analysis, as shown by Shim and coauthors who extended this time from 1 min to 24 h to allow stronger MS2 attachment for their study (Shim et al. 2017).

The similarity of the log_10_ reductions observed between HEEB at 8 kGy and LEEB at 16 kGy for both *G. stearothermophilus* and MS2 on frozen blueberries may be due to several factors, one of them being the difference in the penetration depth of electrons between HEEB and LEEB. The HEEB measured dose is applied to the full depth of the food matrix. The LEEB dose is a surface and subsurface measurement, where the average absorbed dose along the food matrix is depending on the depth dose distribution at a given energy, the matrix density and its chemical composition (Shim et al. 2017). In the current study the beam penetration depth for frozen blueberries was not determined, but in a matrix with water density at 250 keV, the absorbed dose is maximum at the surface while it exponentially drops to zero at approximately 580 µm. If a certain number of microorganisms are beyond the penetration ability of the electrons (at 250 keV) they will not be inactivated. Additionally, it has been hypothesized that lower *D*_10_ value for HEEB versus LEEB treatments may be due to the additional inactivation effect of reactive oxygen species if atmospheres during the treatments contain different amounts of O_2_. Thus, when comparing HEEB and LEEB systems, the focus should not be on comparing the dosimetry measurements. Instead, the impact of achieving the intended microbial reduction and the impact on food quality, such as nutrients and sensory attributes, should be evaluated. The quality of the HEEB and LEEB treated foods in this study were evaluated using sensory attributes. There were no visible differences between HEEB and LEEB treated foods in this study and detailed sensory profiles and chemical properties are described elsewhere (Aisala et al. in preparation).

Based on our results for *G. stearothermophilus* and MS2, a log_10_ reduction of 1.4 can be expected for HAV on frozen blueberries treated by LEEB at 16 kGy, indicating that LEEB processing cannot completely eliminate the risk of viral illness on berries and emphasizes the critical importance of adhering to stringent application of hygiene control systems that limit enteric virus contamination at primary production. In addition, the list of foods allowed for irradiation treatment in the EU currently comprises only dried aromatic herbs, spices and vegetable seasonings and not berries, but this may change in the future, as a legislative evaluation related to the irradiation of food and food ingredients is on-going within the EU (Anonymous 2020).

## Supplementary Information

Below is the link to the electronic supplementary material.

Supplementary file1 (DOCX 56 KB)
