# Supplementary material for: Electron Beam Susceptibility of Enteric Viruses and Surrogate Organisms on Fruit, Seed and Spice Matrices
Source: Food Environ Virol. 2021 Feb 10;13(2):218–28. doi: 10.1007/s12560-021-09463-3 (PMC8116251; doi:10.1007/s12560-021-09463-3)
Supplement: Supplementary file 1 — Supplementary file1 (DOCX 56 KB) [file 12560_2021_9463_MOESM1_ESM.docx]

Supplementary Material

# Supplementary Tables

**Supplementary Table 1**. Raw data used to generate Figure 1.

FD, freeze-dried; p- value, log_10_ reduction different from 0; TI, 95 % prediction interval.

| **Food matrix** | **Microorganism** | **HEEB dose** | **Log_10_ reduction** | **p-value** | **TI** |
| --- | --- | --- | --- | --- | --- |
| Raspberries, FD | HAV | 4kGy | -1,31 | 0,0005 | 0,5114 |
| Raspberries, FD | HAV | 8kGy | -1,57 | 0,0003 | 0,5114 |
| Raspberries, FD | HAV | 16kGy | -1,69 | 0,0002 | 0,5114 |
| Blueberries, frozen | HAV | 4kGy | -0,52 | 0,0249 | 0,6103 |
| Blueberries, frozen | HAV | 8kGy | -1,43 | 0,0002 | 0,6103 |
| Blueberries, frozen | HAV | 16kGy | -2,36 | 0 | 0,6103 |
| Raisins | HAV | 4kGy | -1,37 | 0,0066 | 1,1637 |
| Raisins | HAV | 8kGy | -1,86 | 0,0015 | 1,1637 |
| Raisins | HAV | 16kGy | -3,26 | 0,0001 | 1,1637 |
| Peppercorns | HAV | 4kGy | -0,82 | 0,0003 | ±0,3771 |
| Peppercorns | HAV | 8kGy | -2,1 | 0 | ±0,3771 |
| Peppercorns | HAV | 16kGy | -2,59 | 0 | ±0,3771 |
| Sesame seeds | HAV | 4kGy | -1,22 | 0 | ±0,386 |
| Sesame seeds | HAV | 8kGy | -2,24 | 0 | ±0,386 |
| Sesame seeds | HAV | 16kGy | -3,06 | 0 | ±0,386 |
| Pumpkin seeds | HAV | 4kGy | -0,84 | 0,0001 | ±0,3858 |
| Pumpkin seeds | HAV | 8kGy | -0,73 | 0,0003 | ±0,3858 |
| Pumpkin seeds | HAV | 16kGy | -1,05 | 0 | ±0,3858 |
| Raspberries, FD | MNV | 4kGy | - | - | - |
| Raspberries, FD | MNV | 8kGy | - | - | - |
| Raspberries, FD | MNV | 16kGy | - | - | - |
| Blueberries, frozen | MNV | 4kGy | -0,35 | 0,0229 | ±0,3807 |
| Blueberries, frozen | MNV | 8kGy | -0,76 | 0,0015 | ±0,3807 |
| Blueberries, frozen | MNV | 16kGy | -0.99 | 0,0005 | ±0,3807 |
| Raisins | MNV | 4kGy | -1,35 | 0 | ±0,4251 |
| Raisins | MNV | 8kGy | -1,35 | 0 | ±0,4251 |
| Raisins | MNV | 16kGy | -1,44 | 0 | ±0,4251 |
| Peppercorns | MNV | 4kGy | -0,32 | 0,1246 | ±0,6091 |
| Peppercorns | MNV | 8kGy | -0,26 | 0,1982 | ±0,6091 |
| Peppercorns | MNV | 16kGy | -0,17 | 0,3775 | ±0,6091 |
| Sesame seeds | MNV | 4kGy | -0,96 | 0,0001 | ±0,4023 |
| Sesame seeds | MNV | 8kGy | -1,22 | 0 | ±0,4023 |
| Sesame seeds | MNV | 16kGy | -1,34 | 0 | ±0,4023 |
| Pumpkin seeds | MNV | 4kGy | -0,03 | 0,7334 | ±0,2681 |
| Pumpkin seeds | MNV | 8kGy | -0,09 | 0,3188 | ±0,2681 |
| Pumpkin seeds | MNV | 16kGy | -0,26 | 0,0129 | ±0,2681 |

-: the missing values could not be determined due to technical issues

**Supplementary Table 2.** Raw data used to generate Figure 2.

FD, freeze-dried; p- value, log_10_ reduction different from 0; TI, 95 % prediction interval.

| **Food matrix** | **Microorganism** | **HEEB dose** | **Log_10_ reduction** | **p-value** | **TI** |
| --- | --- | --- | --- | --- | --- |
| Raspberries, FD | *G. stearothermophilus* | 4kGy | -0,68 | 0 | 0,243 |
| Raspberries, FD | *G. stearothermophilus* | 8kGy | -1,62 | 0 | 0,243 |
| Raspberries, FD | *G. stearothermophilus* | 16kGy | -3,94 | 0 | 0,243 |
| Blueberries, frozen | *G. stearothermophilus* | 4kGy | -1,3 | 0 | 0,3756 |
| Blueberries, frozen | *G. stearothermophilus* | 8kGy | -3,33 | 0 | 0,3756 |
| Blueberries, frozen | *G. stearothermophilus* | 16kGy | -5,62 | 0 | 0,3756 |
| Raisins | *G. stearothermophilus* | 4kGy | -0,58 | 0,0002 | 0,2928 |
| Raisins | *G. stearothermophilus* | 8kGy | -1,39 | 0 | 0,2928 |
| Raisins | *G. stearothermophilus* | 16kGy | -3,46 | 0 | 0,2928 |
| Peppercorns | *G. stearothermophilus* | 4kGy | -1,35 | 0 | ±0,1694 |
| Peppercorns | *G. stearothermophilus* | 8kGy | -2,87 | 0 | ±0,1694 |
| Peppercorns | *G. stearothermophilus* | 16kGy | -4,54 | 0 | ±0,1694 |
| Sesame seeds | *G. stearothermophilus* | 4kGy | -1,24 | 0 | ±0,1836 |
| Sesame seeds | *G. stearothermophilus* | 8kGy | -2,87 | 0 | ±0,1836 |
| Sesame seeds | *G. stearothermophilus* | 16kGy | -5,29 | 0 | ±0,1836 |
| Pumpkin seeds | *G. stearothermophilus* | 4kGy | -1,24 | 0 | ±0,3282 |
| Pumpkin seeds | *G. stearothermophilus* | 8kGy | -3,45 | 0 | ±0,3282 |
| Pumpkin seeds | *G. stearothermophilus* | 16kGy | -5,41 | 0 | ±0,3282 |
| Raspberries, FD | MS2 | 4kGy | -0,22 | 0,0219 | 0,2546 |
| Raspberries, FD | MS2 | 8kGy | -0,26 | 0,0104 | 0,2546 |
| Raspberries, FD | MS2 | 16kGy | -0,33 | 0,003 | 0,2546 |
| Blueberries, frozen | MS2 | 4kGy | -0,61 | 0 | 0,1575 |
| Blueberries, frozen | MS2 | 8kGy | -1,15 | 0 | 0,1575 |
| Blueberries, frozen | MS2 | 16kGy | -1,82 | 0 | 0,1575 |
| Raisins | MS2 | 4kGy | -1,21 | 0,0002 | 0,4973 |
| Raisins | MS2 | 8kGy | -2,2 | 0 | 0,4973 |
| Raisins | MS2 | 16kGy | -4,32 | 0 | 0,4973 |
| Peppercorns | MS2 | 4kGy | -0,16 | 0,252 | ±0,428 |
| Peppercorns | MS2 | 8kGy | 0.00 | 0,9953 | ±0,428 |
| Peppercorns | MS2 | 16kGy | -0,76 | 0,0004 | ±0,428 |
| Sesame seeds | MS2 | 4kGy | -0,26 | 0,0001 | ±0,119 |
| Sesame seeds | MS2 | 8kGy | -0,7 | 0 | ±0,119 |
| Sesame seeds | MS2 | 16kGy | -1,36 | 0 | ±0,119 |
| Pumpkin seeds | MS2 | 4kGy | -0,62 | 0,0447 | ±0,849 |
| Pumpkin seeds | MS2 | 8kGy | -0,87 | 0,0101 | ±0,849 |
| Pumpkin seeds | MS2 | 16kGy | -1,48 | 0,0005 | ±0,849 |
| Raspberries, FD | Qβ | 4kGy | -1,09 | 0,0204 | ±1,2308 |
| Raspberries, FD | Qβ | 8kGy | -1,15 | 0,0161 | ±1,2308 |
| Raspberries, FD | Qβ | 16kGy | -1,24 | 0,011 | ±1,2308 |
| Blueberries, frozen | Qβ | 4kGy | 0 | 1 | ±0,3903 |
| Blueberries, frozen | Qβ | 8kGy | -0,94 | 0,0007 | ±0,3903 |
| Blueberries, frozen | Qβ | 16kGy | -1,14 | 0,0003 | ±0,3903 |
| Raisins | Qβ | 4kGy | -0,84 | 0,0008 | ±0,526 |
| Raisins | Qβ | 8kGy | -2,02 | 0 | ±0,526 |
| Raisins | Qβ | 16kGy | -3,23 | 0 | ±0,526 |
| Peppercorns | Qβ | 4kGy | -0,48 | 0,0125 | ±0,4741 |
| Peppercorns | Qβ | 8kGy | -0,74 | 0,0017 | ±0,4741 |
| Peppercorns | Qβ | 16kGy | -0,91 | 0,0006 | ±0,4741 |
| Sesame seeds | Qβ | 4kGy | -0,93 | 0 | ±0,2559 |
| Sesame seeds | Qβ | 8kGy | -1,31 | 0 | ±0,2559 |
| Sesame seeds | Qβ | 16kGy | -2,02 | 0 | ±0,2559 |
| Pumpkin seeds | Qβ | 4kGy | -0,11 | 0,299 | ±0,337 |
| Pumpkin seeds | Qβ | 8kGy | -0,66 | 0,0002 | ±0,337 |
| Pumpkin seeds | Qβ | 16kGy | -1,35 | 0 | ±0,337 |

-: the missing values could not be determined due to technical issues

**Supplementary Table 3**. Significance (S) – Non-significance (NS) of mean MS2 log_10_ reductions and overlap of prediction intervals *versus* *G. stearothermophilus*, HAV and Qβ.

| **MS2 *versus*** | **Food matrix** | **HEEB dose** | **S / NS?** | **Overlap of**  **prediction intervals** |
| --- | --- | --- | --- | --- |
| *G. stearothermophilus* | Blueberries, frozen | 4kGy | S – Lower | No |
| *G. stearothermophilus* | Blueberries, frozen | 8kGy | S – Lower | No |
| *G. stearothermophilus* | Blueberries, frozen | 16kGy | S – Lower | No |
| *G. stearothermophilus* | Raspberries, FD | 4kGy | S – Lower | Yes |
| *G. stearothermophilus* | Raspberries, FD | 8kGy | S – Lower | No |
| *G. stearothermophilus* | Raspberries, FD | 16kGy | S – Lower | No |
| *G. stearothermophilus* | Raisins | 4kGy | S – Lower | Yes |
| *G. stearothermophilus* | Raisins | 8kGy | S – Lower | No |
| *G. stearothermophilus* | Raisins | 16kGy | S – Lower | No |
| *G. stearothermophilus* | Peppercorns | 4kGy | S – Lower | No |
| *G. stearothermophilus* | Peppercorns | 8kGy | S – Lower | No |
| *G. stearothermophilus* | Peppercorns | 16kGy | S – Lower | No |
| *G. stearothermophilus* | Sesame seeds | 4kGy | S – Lower | No |
| *G. stearothermophilus* | Sesame seeds | 8kGy | S – Lower | No |
| *G. stearothermophilus* | Sesame seeds | 16kGy | S – Lower | No |
| *G. stearothermophilus* | Pumpkin seeds | 4kGy | S – Lower | Yes |
| *G. stearothermophilus* | Pumpkin seeds | 8kGy | S – Lower | No |
| *G. stearothermophilus* | Pumpkin seeds | 16kGy | S – Lower | No |
| HAV | Blueberries, frozen | 4kGy | NS – Higher | Yes |
| HAV | Blueberries, frozen | 8kGy | NS – Lower | Yes |
| HAV | Blueberries, frozen | 16kGy | S – Lower | * |
| HAV | Raspberries, FD | 4kGy | S – Lower | No |
| HAV | Raspberries, FD | 8kGy | S – Lower | No |
| HAV | Raspberries, FD | 16kGy | S – Lower | No |
| HAV | Raisins | 4kGy | N – Lower | Yes |
| HAV | Raisins | 8kGy | N – Higher | Yes |
| HAV | Raisins | 16kGy | * | * |
| HAV | Peppercorns | 4kGy | S – Lower | Yes |
| HAV | Peppercorns | 8kGy | S – Lower | No |
| HAV | Peppercorns | 16kGy | S – Lower | No |
| HAV | Sesame seeds | 4kGy | S – Lower | No |
| HAV | Sesame seeds | 8kGy | S – Lower | No |
| HAV | Sesame seeds | 16kGy | S – Lower | No |
| HAV | Pumpkin seeds | 4kGy | N – Lower | Yes |
| HAV | Pumpkin seeds | 8kGy | N – Higher | Yes |
| HAV | Pumpkin seeds | 16kGy | N – Higher | Yes |
| Qβ | Blueberries, frozen | 4kGy | S – Higher | No |
| Qβ | Blueberries, frozen | 8kGy | * | * |
| Qβ | Blueberries, frozen | 16kGy | * | * |
| Qβ | Raspberries, FD | 4kGy | S – Lower | Yes |
| Qβ | Raspberries, FD | 8kGy | S – Lower | Yes |
| Qβ | Raspberries, FD | 16kGy | S - Lower | Yes |
| Qβ | Raisins | 4kGy | S – Higher | Yes |
| Qβ | Raisins | 8kGy | NS – Higher | Yes |
| Qβ | Raisins | 16kGy | S – Higher | No |
| Qβ | Peppercorns | 4kGy | S – Lower | Yes |
| Qβ | Peppercorns | 8kGy | S – Lower | * |
| Qβ | Peppercorns | 16kGy | NS – Lower | Yes |
| Qβ | Sesame seeds | 4kGy | S – Lower | No |
| Qβ | Sesame seeds | 8kGy | S – Lower | No |
| Qβ | Sesame seeds | 16kGy | S – Lower | No |
| Qβ | Pumpkin seeds | 4kGy | S – Higher | Yes |
| Qβ | Pumpkin seeds | 8kGy | NS – Higher | Yes |
| Qβ | Pumpkin seeds | 16kGy | NS – Higher | Yes |

* Maximum measurable log_10_ reduction reached
